# Analysis of the Consumption of Caffeinated Energy Drinks among Polish Adolescents

**DOI:** 10.3390/ijerph120707910

**Published:** 2015-07-10

**Authors:** Dariusz Nowak, Artur Jasionowski

**Affiliations:** 1 Department of Nutrition and Dietetics, Faculty of Health Sciences, Nicolaus Copernicus University in Toruń, Ludwik Rydygier Collegium Medicum in Bydgoszcz, Dębowa 3, Bydgoszcz 85-626, Poland; 2 Department of Theoretical Foundations of Biomedical Sciences and Medical Informatics, Faculty of Pharmacy, Nicolaus Copernicus University in Toruń, Ludwik Rydygier Collegium Medicum in Bydgoszcz, Dębowa 3, Bydgoszcz 85-626, Poland; E-Mail: artuja@wp.pl

**Keywords:** adolescents, caffeine, dependence, energy drink, overdose

## Abstract

Background: Energy drinks (EDs) are extremely popular among adults and adolescents. Regular intake of EDs may lead to an overdose of caffeine, loss of bone mass, overweight, hypertension and, in older age, osteoporosis and cardiovascular diseases. Some people mix EDs with alcohol, which adversely affects their health. The objective of this study was to analyze the consumption of EDs by adolescents. Methods: The study consisted of a questionnaire surveying amounts of drinks, preferences and product awareness among younger consumers. The study was carried out in junior and senior high schools in Poland (n = 2629). Results: EDs were consumed by 67% of students (quite frequently by 16%). Students who practiced sports were more willing to drink EDs. Also, boys drank them more often than girls. When selecting a particular ED, young people looked at the taste, price and effect. Most respondents consumed one ED (250 mL) daily, although there were individuals consuming two or more drinks daily. Most respondents knew the ingredients of EDs, and 24% admitted to mixing EDs with alcohol. Conclusions: EDs are extremely popular among adolescents. Young people drinking EDs every day are potentially at risk of taking an overdose of caffeine.

## 1. Introduction

The first energy drinks (EDs) appeared in Europe and Asia in 1960, but they did not gain popularity until the launch of a new drink called Red Bull (in 1987 in Austria and in 1997 in the USA). Since then, consumption of EDs has been growing worldwide. In 2006, the global consumption of these beverages increased by 17% compared to the previous year [1,2]. In 2014, the energy drink Red Bull alone (43% of the global market) sold 5.612 billion cans, and the growth in its sales was 4.2% *vs.* the year 2013 [3]. The largest consumption of EDs was noted in such countries as the United States of America, Vietnam, Cuba, the United Kingdom, Thailand, Mexico, Australia, Germany, Poland and Saudi Arabia [4]. The highest increase in sales of Red Bull (from 2013 to 2014) was recorded in Turkey (+33%), India (+32%), the RSA (+31%), Russia (+18%), Japan (+11%) [3]. In the UK alone, the annual consumption of EDs increased from 4.4 litres per person in 2007 to 9.4 litres per person in 2014. There are currently hundreds of brands of EDs available across the world, and producers have adopted new sales strategies. Now they address these drinks to new groups of consumers (elderly people) and new occasions (for breakfast) or they inform consumers about new functional aspects (improved memory and accelerated metabolism), often implying that these drinks are healthier and more natural.

Some EDs are offered in unusual flavors (fruity, mint) and packaging (bottles emitting fluorescent light in the dark) [5]. Energy drinks can contain more than 15 ingredients, but the essential components come in five categories: (1) caffeine; (2) a sweetener of some kind (usually sugar); (3) one or more amino acids (most often taurine but sometimes L-carnitine); (4) vitamins B and (5) one or more plant/herbal extracts such as ginko biloba, guarana, ginseng, milk thistle *etc*. Some of the ingredients raise doubts because energy drinks are also cosumed by children and teenagers. A can or a bottle of ED contains from 50 to 505 mg of caffeine, depending on the capacity (generally, 80–141 mg per 250 mL can), which equals, or even exceeds, the caffeine content of a cup of coffee, typically ranging between 77 and 150 mg [1,6]. Energy drinks often contain additional amounts of caffeine in other ingredients, such as kola nuts, guarane, or yerba mate [7]. Beverages with caffeine are regularly drunk by children, which may lead to a caffeine overdose, loss of osseous mass (calcium binding), insomnia and overweight (sugar); in later years, excessive consumption may be responsible for osteoporosis and cardiovascular diseases [8]. Energy drinks can contain three-fold more caffeine than sweetened beverages, and even moderate quantities of caffeine (200–350 mg) increase arterial blood pressure [8]. Other ingredients of EDs raise less controversy. Apart from caffeine, a 250 mL can of ED contains 4000 mg/L of taurine and 2400 mg/L of D-glucurono-γ-lactone. Assuming an average consumption at 0.5 can per person in 2003, the SCF (Scientific Committe on Food) has calculated that the average consumption of taurine is 500 mg in addition to 300 mg of D-glucurono-γ-lactone. D-glucurono-γ-lactone ingested p.o. is quickly absorbed by humans, metabolized and excreted as glucuric acid. Therefore, the SFC has concluded that, in light of contemporary research, there is no risk to human health due to the taurine and D-glucurono-γ-lactone found in energy drinks at their current concentrations [9].

Negative consequences of drinking EDs can be due to the toxicity of certain ingredients, mixing of EDs with alcohol and drugs, or accompanying physical exertion [2,6]. This risk is worse in certain populations, including children and teenagers, pregnant women, people with underlying cardiac conditions and other caffeine-sensitive individuals. Many adolescents and young adults drink large quantities of EDs with alcohol. In fact, as many as 25%–40% [6,10], or even 60% [11] of young people admit to it. Deaths associated with consumption of energy drinks have been recorded in Australia, Ireland and Sweden [1,12]. However, many fatal cases have been attributed to the mixing of energy drink with alcohol. The consequences might include impaired cognitive functions, weaker symptoms of alcoholic intoxication, masked effects of alcohol and consequently a higher risk of developing alcohol addition [13,14,15].

Considering the growing popularity of EDs among young people all over the world and the health risks caused by their excessive consumption, it is worthwhile to conduct research in this field. Therefore, the objective of this study was to analyze the consumption of energy drinks by adolescents in the context of amounts of drinks, preferences and the awareness among younger consumers about the composition of such beverages and possible risks.

The results of the study will shed the light on the problem of excessive energy drink consumption among young Poles. Similar studies in Poland are limited and do not cover caffeine intake from other sources. In the context of the growing worldwide consumption of energy drinks, this research will enable us to assess the volume of EDs consumed by adolescents as well as the awareness and motivation of consumers who choose such drinks. The results can assist in developing legal solutions and educational activities.

## 2. Materials and Methods

### 2.1. Subjects

The investigations were carried out in 2012–2013, in junior and senior high schools located in the four largest towns in the Province of Kujawy and Pomorze in Poland: Bydgoszcz, Toruń, Włocławek and Grudziądz. The selection of schools in each location was random, aided by the random number formula (an MS Excel application).

The adolescents participated in the survey voluntarily, having obtained consent from their legal guardians and the school head teacher. In total, 2629 people completed the survey. The research was accepted by the University Bioethical Committee (KB 585/2012) and completed in compliance with the Helsinki Declaration.

### 2.2. Assessments

A survey method was applied. During a designated interval at a lesson, trained assistants (undergraduate college students) handed out copies of a questionnaire, designed by the research authors, to be completed by each student individually and voluntarily. The respondents answered 31 questions and deposited the questionnaires in a sealed box. Questions 1 and 2 pertained to some demographic data, such as the age and gender of a respondent. Questions 3 and 4 asked the respondents to give their body weight/height and place of residence (some pupils commute to their schools from smaller localities). Questions 5 to 7 concerned sports, frequency of sporting activities and practiced disciplines. Question 8 was a screening one, serving to identify respondents who consumed ED (“Do you drink energy drinks?”). The respondents who gave a “no” answer were instructed to omit certain questions and pass over to Question 19, which assessed knowledge of the composition of energy drinks and their effects. If a respondent gave a positive answer to Question 8, he or she was instructed to continue answering the following questions, to assess the type of energy drink consumed (3 brands at the most; Question 9), frequency of drinking such beverages (every day, a few times a week, once a week, once a year, Question 10). Next, the pupils who drank EDs were asked how much they drank daily, in terms of the number of 250-mL cans/bottles (Question 11) and a portion of energy drink they typically had (Question 12). Questions 13–16 were about preferences concerning the packaging (can, bottle, it does not matter), price, place of purchase (hypermarket/supermarket, corner shop, petrol station, newsagent’s, school shop/cafeteria, restaurant, pub, vending machine) and time of day when they usually consumed an energy drink (home, school, on the way to school/home, free time). Question 17 asked: “Who most often buys energy drinks for you?” Next, the respondents were inquired about what motivated their choice of which energy drink to buy (Question 18). They could point to more than one of the given answers (price, amount, flavour, effect, packaging, advertisements or friends’ recommendation, fashion, availability, other). Question 19 was answered by both groups, those who consumed energy drinks and those who did not. They were asked if they knew the composition of EDs (yes/no), and then to point to ingredients. Question 21 was meant to find out reasons for drinking energy drinks (before and after physical or mental effort, when I feel tired, when I feel sleepy, when I feel like having something to drink, a party, no particular reason). Next, they were asked how they felt after having an energy drink (I feel normal, bad, overexcited, tired, first overexcited and then tired, like after drinking alcohol) and if they had ever suffered any discomfort after having an energy drink. Question 24 was about mixing energy drinks with alcohol, and the following question inquired about any bad side-effects of mixing EDs with alcohol. Question 26 tested the respondents’ awareness of the harmfulness of energy drinks. There were three possible replies “They are harmful”, “They are harmless”, “It depends on how much you drink”. The last five questions were about consumption (frequency and amounts) of other beverages containing caffeine, such as coffee, tea or cola-type beverages.

### 2.3. Statistical Analyses

Statistical analysis of the results was supported by the software programme Statistica (ver. 9.1). The Pearson χ^2^ test was employed to assess differences in the distribution of frequency of replies. The level of significance *p* = 0.05 was maintained at all the statistical tests.

## 3. Results

The questionnaire was answered by 2629 respondents (1481 female, 1148 male participants), aged 12–20 (average age 15.8 years), BMI 19.8 ± 1.2 kg/m^2^. Most respondents lived in town (84%), over half of which (51%) lived in a town with the population of over 200,000. 16% lived in the country. The respondents attended junior high schools (55%) and senior high schools (45%). Most (76%) declared that they participated in sports.

Energy drinks were consumed by 1756 students (67%). EDs were significantly more often drunk by boys than girls (*p* < 0.01). In the group of 1148 of boys, EDs were consumed by nearly 75% of respondents, whereas only 61% of the girls among 1481 answering the survey consumed EDs. Those who declared that they played some sports consumed energy drinks more often (77%). Among those who did not play sports, only 23% drank EDs. Place of residence (bigger and smaller towns) did not significantly affect (*p* = 0.11) the consumption of energy drinks by adolescents.

With respect to the frequency of consumption, most of the respondents had an energy drink once a month (20% replies). Over 16% of teenagers said they had energy drinks quite often (daily, a few times a week, once a week) (Table 1). Over 2% of the adolescents surveyed consumed energy drinks every day.

**Table 1:** Consumption of energy drinks by adolescents.

Sex	Female n = 1481	Male n = 1148	Total n = 2629	*p*-Value
Consumption of energy drinks	n (%)	n (%)	n (%)	
	900 (60.8) *	856 (74.6) **	1756 (66.8) ***	<0.01
Daily (%)	18 (1.2)	37 (3.2)	55 (2.1)	
Several times/week (%)	74 (5.0)	137 (11.9)	211 (8.0)	
1/week	65 (4.4)	94 (8.1)	159 (6.05)	
Several times/month	210 (14.2)	224 (19.4)	434 (16.5)	
1/month	295 (19.9)	231 (20.1)	526 (20.0)	
1/year	238 (16.1)	137 (11.9)	375 (14.3)	

* Female = 1481; ** Male = 1148; *** n = 2629.

The type of school had some effect on the consumption of energy drinks. Significantly more EDs were consumed by students from junior than from senior high schools (*p* = 0.017). The most popular brands of energy drinks were Tiger (56%), Red Bull (47%), Mountain Dew (42%) and Burn (30%). When the respondents were asked about the number of 250-mL cans/bottles drunk daily, most declared drinking 1 portion a day (44%). Having 2 portions a day was declared by 12% of students, while 3 or 4 drinks were consumed by 2 and 3% of respondents, respectively. The most popular type of container was a can (61%), followed by a PET bottle (25%). Most students (52%) chose an energy drink in the price range of 2–4 PLN (0.5–1 EUR). Some respondents (11%) bought less expensive (<0.5 EUR), and some (22%) chose more expensive (>1 EUR) drinks, while for the remaining students (15%) the price did not matter. The most popular place of purchase was a hypermarket (53%). The respondents most often drank EDs (34%) at home, on a day off school (40%). The next question concerned determinants influencing the choice of ED. The selection of these and the following determinants were significant (*p* < 0.05). Most respondents chose energy drinks because of their taste (63%), price (32%) or effect (24%). The amount of ED per can/bottle, availability of a brand, packaging or advertising were important only at a later stage of choice making (Table 2). 

**Table 2:** Determinants influencing the purchase of energy drink.

Determinant	Girls (%)	Boys (%)	Total (%)
Price	27.3	36.3	32.5 *
Amount	6.7	13.6	10.3 *
Flavour	69.8	53.7	63.4 *
Effect	21.1	25.1	23.6
Package	6.8	4.9	6.0
Advertisement	4.4	4.9	4.8
Fashion	2.7	2.9	2.9
Availability	7.8	7.7	7.8

n = 1756 (girls = 900; boys = 856), * *p* < 0.05.

In the assessment of the respondents knowledge about the ingredients of energy drinks, caffeine (about 90%), sugar (78%) and taurine (63%) were most often indicated (Table 3). Some respondents also selected the answer “much energy” (over 28%), a phrase often used in advertisements of energy drinks. Others pointed to fat, protein, vitamins and minerals.

**Table 3:** Knowledge of the ingredients of energy drinks.

Ingredient	Girls (%)	Boys (%)	Total (%)
Caffeine	89.8	89.5	89.7
Sugar	79.4	77.2	78.4
Taurine	55.6	73.3	63.3 *
Much energy	27.9	29.4	28.6
Fat	16.7	16.3	16.5
Vitamins	9.6	20.7	14.5 *
Minerals	9.0	13.9	11.1 *
Protein	5.7	13.8	9.2 *
Other	3.4	6.6	4.8 *

n = 2629 (girls = 1481; boys = 1148), * *p* < 0.05.

Adolescents most often drank energy drinks without any particular reason (21%) or when they felt tired (18%). Some had them before or after physical effort (13% and 10%, respectively). Some also drank them before and after mental effort (5% and 1%, respectively). Some students (12%) consumed EDs when they felt thirsty. Nearly 10% of teenagers had energy drinks at parties.

Quite a large group of adolescents believed that energy drinks were bad for their health (36%) or said it depended on the amount consumed (58%). Just 6% of respondents claimed energy drinks had no adverse influence on health. On the other hand, 7% (195) admitted to feeling some discomfort after drinking an ED. This group was composed of 109 girls and 86 boys. The most common health problems were: stomach ache (46%), anxiety and heart palpitations (15%), and nausea and vomiting (15%). Most respondents felt normal (61%). Nearly 27% of respondents felt overexcited after drinking an ED, and about 8% felt first overexcited and then tired.

Many respondents admitted to mixing energy drinks with alcohol. This group comprised 24% of students from both types of schools. Gender did not have a significant influence (*p* = 0.204) on the preference for combining energy drinks with alcohol (Table 4). On the other hand, the type of school significantly affected this reply (*p* < 0.01). More students from senior high schools (37%) than from junior high schools (14%) mixed EDs with alcohol (Table 4).

**Table 4:** Number of students mixing energy drinks with alcohol.

Sex	No Mixing	Mixing	*p*-Value
Female	1120	358	0.204
Male	868	277	
Senior high school	741	432	<0.01
Junior high school	1247	203	
***Total***	***1988***	***635***	

The age of the respondents correlated significantly (*p* < 0.01) with the habit of mixing energy drinks with alcohol. This fact was admitted mostly by 17 (34%), 18 (44%), 19 (49%) and 20-year-olds (71%). Students from the first and second grade of junior high schools (aged 13–14) were the ones who least frequently mixed energy drinks with alcohol (7% and 10% of respondents, respectively).

The town where the study was completed had some influence on this aspect. The respondents from schools in Toruń and Włocławek drank EDs mixed with alcohol most often (Table 5). On the other hand, young people attending schools in Grudziądz least often mixed energy drinks with alcohol (Table 5). Consequently, the size of a town did matter significantly.

**Table 5:** Consumption of energy drinks mixed with alcohol in different cities.

City	No Mixing n (%)	Mixing n (%)	Total n	*p*-Value
Bydgoszcz	1209 (78)	336 (22)	1545	<0.01
Toruń	445 (70)	187 (30)	632	
Włocławek	247 (72)	96 (28)	343	
Grudziądz	87 (85)	16 (15)	102	

The respondents who admitted that they combined consumption of EDs and alcohol were asked if they experienced “any disorders” afterwards and what type of health problems they had. In total, 4.4% (n = 116) of the respondents, consisting of 5.2% boys (60/1148) and 3.8% girls (56/1481), reported mainly vomiting and other problems such as nausea and headache, dizziness, heart palpitations and stomach ache. Apart from energy drinks, respondents consumed other beverages containing caffeine, namely coffee (43%, of which half drank natural coffee), black tea (67%) and green tea (47%), cola-type beverages (85%). Excessive consumption of energy drinks in combination with coffee, tea or sodas potentially poses a risk of overdosing on caffeine. Therefore, in the subgroup of adolescents who consumed EDs daily, other sources of caffeine intake (coffee, black tea, green tea, cola-type beverages) were estimated (Table 6).

**Table 6:** Caffeine intake in the subgroup of adolescents who consume EDs every day.

Sources of Caffeine	n	Range of Daily Intake of Caffeine (mg/person)	Average Daily Intake of Caffeine (mg/person)
EDs	55	80–320	205.1
Coffee	24	64–168	122.5
Black tea	36	42–210	87.1
Green tea	25	40–200	48.7
Cola-type beverages	45	25–125	55.0
Total			518.4

The results showed that the average daily intake of caffeine in this subgroup was 518.4 mg/day, including 205.1 mg from EDs. Some students declared consumption of 4 EDs per day (above 300 mg of caffeine) beside drinking coffee, tea and cola-type beverages. In the subgroup of adolescents who consumed EDs every day more than half admitted to mixing EDs with alcohol.

## 4. Discussion

The reported research showed that 67% of students in the age range of 12–20 years (the average age 15.8 years) consumed energy drinks (EDs). Over 16% of respondents (composed of 268 male and 157 female respondents) declared they consumed energy drinks quite frequently. Results of other studies confirm frequent consumption of EDs by young people. It has been demonstrated, for example, that over half of the cosumers are children (<12 years old), teenagers (12–18 years old) and young people (19–25 years old) [7]. Regular consumption of energy drinks was indicated by 28% to 31% of teenagers aged 12 to 17 [16] and 34% of persons aged 18–24 [17]. Energy drinks are consumed often by 10% and rarely by 46% to 51% of young people [18]. In the current study, 16% of students stated that they often had EDs (every day, a few times weekly, once a week). Recently, in the published report of EFSA (16 EU countries, over 52,000 participants) consumption of energy drinks was reported by approximately 68% of adolescents [19]. Among these, about 12% were “high chronic” consumers, with an average consumption of 7 litres per month. These results were similar to ours. We were able to distinguish the determining factors for making a choice of a particular energy drink. Most of the young consumers choose an energy drink based on taste, while the price and effect of the drink were secondary. In other studies, taste was also an important reason for consuming EDs [20]. Our study showed that one in five respondents drank an ED for no particular reason or when they felt tired. One in four had it before, or after, physical effort. There was also a group of respondents (12%) who had energy drinks when they felt thirsty, although it is healthier and better to quench thirst with tap water or still mineral water. Caffeine has a dehydrating effect, which is why an energy drink cannot be recommended after physical effort. In addition to the above, other researchers pointed to such reasons for drinking EDs as: studying for exams, completing projects, increasing mental alertness and enhancing energy levels [20]. In our study, respondents most frequently chose the following two brands of EDs: Tiger (56%) and Red Bull (47%). For comparison, in another investigation, respondents preferred mostly Red Bull (47%) with Monster as their second choice (40%) [20]. Our study showed that people active in sports more often consumed EDs. Water, special nutrition beverages (isotonic drinks) or a supplement which will provide the body with the liquids and minerals lost during the effort are better choices. Some of our respondents had energy drinks before undertaking physical effort, probably hoping to improve performance. This is a common motivation found in other investigations. In Canada, for example, among 16,000 interviewed children and teenagers (aged 11–18), 27% had an intake of caffeine to potentially improve their exercise capacity, and 13% were encouraged to do so by their coaches. Similar results were reported for children and teenagers in the USA [8]. 28% of our respondents claimed that energy drinks gave them a boost of energy, although the calorific value of such a drink is no different from any other type of soda. In another study completed on college students, it was reported that 54% of the respondents had energy drink to gain energy [21]. The percentage was even higher (65%) in yet another study [22]. The interviewed young people knew the basic ingredients of energy drinks (caffeine, sugar, turine) and agreed that they might be harmful. On the other hand, only 7% admitted to experiencing some health disorders after drinking ED. The most common problems reported were stomachache (about 50% replies), and—to a much lesser degree—overexcitement, heart palpitations or vomiting. In a study on college students, most energy drink consumers complained of headaches (22%) and heart palpitations (19%) [22]. The most frequently reported side-effects of caffeine poisoning are nausea/vomiting (56%), bradycardia (44%), hypertension (100%), anxiety/spasms (67%), dizzy head (44%), chest pain (11%) and bilateral paresia (11%) [1,23]. Of the 5448 reported cases of caffeine overdose in the USA in 2007, as many as 46% pertained to patients under 19 years of age [7].

A worryingly large group (24%) admitted to mixing energy drinks with alcohol. Most of those respondents were students attending the final grade of senior high schools. Nearly half of the group of persons aged 17–18 declared they that drank ED with alcohol. Among the youngest respondents (13–14 years old), this problem affected no more than 10%. A report published by EFSA [19] showed that a larger number (about 53%) of adolescents mixed energy drinks with alcohol. Bonar *et al.* reported an even higher percentage of youth drinking EDs with alcohol (about 60%) [11], while in other studies conducted on older subjects (college students), 54% [22] or 19% [21] of respondents admitted to mixing EDs with alcohol. Meanwhile, in a study conducted in the USA, 24.8% of students admitted to mixing EDs with alcohol [10], a percentage similar to ours. The habit of mixing energy drinks and alcohol has become very popular among students. There are 400 known recipes for cocktails with energy drinks [24]. However, drinking an ED (Red Bull) with vodka has been reported to impair physical coordination [25]. Other negative consequences are dehydration, insomnia, headaches, anxiety, nose bleeding and vomiting, heart arrhythmia or even death [12]. In our survey, only 4% of the respondents admitted to experiencing any disorders due to the consumption of energy drinks with alcohol. The most frequent health problems were vomiting, nausea and severe headaches. In other studies, respondents mostly reported feeling jittery (71%) and having trouble sleeping (46%) [11]. Moreover, there is a growing body of evidence suggesting that drinking caffeinated alcoholic beverages can be riskier than drinking pure alcoholic beverages [26]. A recent study has demonstrated that students who drank alcohol mixed with energy drinks had more accidents (drinking and driving, minor injuries, rapes) than people who drank pure alcohol [17]. Recent studies have demonstrated that mixing EDs with alcohol by young people may lead to the highest rates of risk behaviors (drug use, risky sexual behaviors, drinking and driving, increasing alcohol abuse and smoking) [11,14,27,28].

Research shows that the consumption of caffeine by children and teenagers has been growing. Its average consumption in the age group of 5 to 18 was 38 mg/day in 1982 [29]. A subsequent study, conducted in the 1990s in the USA, showed an average caffeine intake by teenagers between 12 and 17 years of 69.5 mg/day, which was slightly less than contained in a cup of coffee [30]. The principal source of caffeine in children’s and teenagers’ diets are beverages, namely cola-type drinks, unlike in the diet of adults, who mostly (71%) obtain caffeine from coffee [30]. This was confirmed by our study, in which most of the young respondents, who readily consumed other sources of caffeine apart from energy drinks, admitted to drinking cola-type beverages (85%). Almost half of the respondents also enjoyed drinking coffee and green tea, whilst nearly 70% drank black tea. It must be remembered that at such a young age, the demonstrated preference for various sources of caffeine, in combination with the consumption of energy drinks, can lead to health disorders and complications, due to excessive caffeine intake. The estimated average daily intake of caffeine in the subgroup of adolescents who consumed EDs every day was above 500 mg/day. It should be emphasized that even moderate amounts of caffeine (more than 200–350 mg) cause an increase in blood pressure [8] and are potentially harmful.

## 5. Conclusions

Energy drinks are extremely popular among adolescents. In our study, 67% of the 2629 students surveyed consumed EDs, with 16% drinking them quite often (every day, a few times a week, once a week). Age (younger rather than older respondents drink more EDs), gender (boys rather than girls) and active sports participation affect the likelihood of drinking energy drinks. Older students more often drank EDs mixed with alcohol (34% to 49% in the age group of 17 to 19 years *vs.* 7% to 10% in the age group of 13 to 14 years).

This investigation concludes that adolescents most often drank canned energy drinks, bought in hypermarkets, in the price range of 2–4 PLN (0.5–1 EUR). When selecting a brand, students most often looked at the taste, price and effect. Apart from EDs, young people also like other sources of caffeine, including cola-type beverages, tea and coffee. Among the respondents consuming energy drinks every day (especially more than once daily), there is a high risk of consuming excessive amounts of caffeine, which can pose a health threat in the long term. More screening tests should be conducted on the consumption of EDs and other sources of caffeine in the diets of children and adolescents. It is also necessary to focus on the problem of mixing energy drinks with alcohol (one in four respondents admitted to it) and commence educational campaigns in order to inform young people about the harmfulness of such consumption.
